# Suicide Screening Tools for Pediatric Emergency Department Patients: A Systematic Review

**DOI:** 10.3389/fpsyt.2022.916731

**Published:** 2022-07-12

**Authors:** Amanda Scudder, Richard Rosin, Becky Baltich Nelson, Edwin D. Boudreaux, Celine Larkin

**Affiliations:** ^1^New York Medical College, New York, NY, United States; ^2^University of Massachusetts Chan Medical School, Worcester, MA, United States

**Keywords:** suicide, youth, pediatric, emergency, screening

## Abstract

**Background:**

According to the Centers for Disease Control and Prevention, suicidality and suicidal behavior among youth continues to increase significantly each year. Many of those who die by suicide interact with health services in the year before death. This systematic review sought to identify and describe empirically tested screening tools for suicidality in youth presenting to Emergency Departments (ED).

**Objective:**

(1) To identify and compare existing tools used to screen for suicidality in children and adolescents who present to the ED and (2) to ascertain the prevalence of suicidality in pediatric populations found with these tools.

**Methods:**

We searched Ovid Medline, CINAHL, Scopus, and Cochrane databases for primary research studies that identified and evaluated screening tools for suicide risk in pediatric ED patients. A total of 7,597 publications published before August 25, 2021 met search criteria and were screened by two independent reviewers based on our inclusion and exclusion criteria, with any conflicts resolved via consensus meetings or an independent reviewer. A total of 110 papers were selected for full text review, of which 67 were excluded upon further inspection. Covidence was used to extract and synthesize results.

**Results:**

43 articles were eligible for inclusion. Most studies (*n* = 33) took place in general pediatric EDs; the quality was generally high. Patients ranged from 4-24 years old, with most screening tested in patients 12 years and older. The most researched tools were the Ask-Suicide Screening Questions (ASQ) (*n* = 15), Columbia-Suicide Severity Rating Scale (C-SSRS) (*n* = 12), Suicidal Ideation Questionnaire (SIQ) (*n* = 11), and the Risk of Suicide Questionnaire (RSQ) (*n* = 7). Where screening was applied to all patients, about one-fifth of pediatric ED patients screened positive; where suicide screening was applied to psychiatric patients only, over half screened positive. Positive screens were more likely to be female and older than negative screens and they were more likely to be assessed and admitted.

**Conclusion:**

Several validated screening tools exist for the purpose of screening pediatric populations in EDs for suicidality. Such tools may help to support early detection and appropriate intervention for youth at risk of suicide.

**Systematic Review Registration:**

https://www.crd.york.ac.uk/prospero/display_record.php?RecordID=276328, identifier: 276328

## Introduction

Nearly one in every five young people have seriously considered suicide, and almost 10% reported having attempted suicide (1). According to the Centers for Disease Control and Prevention (CDC), suicide among individuals ages 10-24 has increased in prevalence every year from 2007 to 2018, with a total increase of nearly 60% in that time (2). Youth are especially vulnerable, given their stage of development, and their decreased autonomy in scheduling and presenting for medical care compared to adults.

Existing research shows that many individuals who die by suicide consult health services prior to their death: 9% on the day of death, 34% during the week prior, and 61% in the month before their death (3); emergency department visits are particularly prevalent among suicide decedents (4). Therefore health services represent a key venue for the detection and management of suicide risk in young people. Screening patients is an efficient way of identifying potential suicide risk in youth in healthcare settings.

The goal of screening is to identify the subset of patients displaying non-negligible suicide risk, which is then assessed further by a clinician (5). Screening may be done “universally” with all patients regardless of presenting complaint or “selectively” focused on patients with an increased prevalence of suicidality, such as psychiatric patients (6). The screening modality may be verbal, paper-based, or via a computer or tablet; an ideal suicide screening tool is brief, feasible to administer, has good psychometric properties, and is sensitive enough to detect non-negligible risk (5).

Evidence suggests that screening for suicidality does not increase suicide risk (7); these findings hold for youth (8, 9). Moreover, screening for suicidality in acute care settings appears to be acceptable to youth and parents (10). Based on the now substantial evidence base for screening and the recent increase in youth suicidality, there is mounting support for the implementation of suicide screening as a part of routine healthcare for youth (11). Currently, there is no standard of care for screening youth for suicidality in emergency department (ED) settings, which tend to serve as the frontline of acute healthcare. This systematic review aimed to identify and describe empirically tested screening tools for suicidality in youth presenting to Emergency Departments (ED).

## Methods

This study was registered with PROSPERO, the international prospective register of systematic reviews (registration number # CRD42022276328) and followed the guidelines set forth by the Preferred Reporting Items for Systematic Reviews and Meta-Analyses (PRISMA) statement (12).

### Eligibility Criteria

To be eligible, articles had to meet the following inclusion criteria: (1) involve suicide-related screening tools that were empirically applied to the ED patient population; (2) include primary data collection from ED patients; (3) have tested the screening tool on the pediatric population, which we defined as samples that were mostly under the age of 21 years old; (4) have tested the screening tool on a research population that includes both suicidal and not suicidal patients.

Exclusion criteria were articles that were: (1) about screening tools that have not yet been applied to patients; (2) narrative and systematic reviews; (3) not peer reviewed; (4) about screening tools that only apply to adult-only populations; (5) focused only on other care settings, such as prehospital, inpatient, and outpatient settings; (6) focused on screening for polymorphisms or other blood screening that could serve as a marker of increased risk; (7) solely focused on individual risk factors of suicidality that are not compiled into a screening tool; (8) not available in the English language; and (9) focused on suicide attempters only (because we were focused on screening tools to detect suicide risk, not to further stratify or measure known suicide risk).

### Information Sources and Search Strategy

A comprehensive literature search was conducted by a medical librarian on August 25th, 2021, using the following bibliographic databases from inception: Ovid MEDLINE® (ALL-1946 to Present); CINAHL with Full Text (EBSCO); Cochrane Library (Wiley); Ovid PsycInfo (1967 to Present); and Scopus (Elsevier). No article type, date, or language restrictions were included in the search. Controlled vocabulary and keywords for self-injurious behaviors, smartphones, and mobile applications were included in the search. The full Ovid MEDLINE search strategy is available in (Supplementary Table 1).

### Study Selection Process

The 10,207 results produced from the database searches were imported into Covidence, a systematic review management system, and were de-duplicated. The remaining 6,584 citations were screened by title and abstract against predetermined, aforementioned inclusion and exclusion criteria by two independent reviewers, with discrepancies resolved by consensus or a third reviewer.

The remaining full-text articles were screened against predetermined inclusion and exclusion criteria by two independent reviewers, with discrepancies resolved by consensus or a third reviewer.

Reference lists and forward citations for included articles were gathered and deduplicated, producing 1,013 additional citations for screening, for a total of 11,220 studies imported for screening. In total, there were 110 articles selected for full-text review, 43 of which met inclusion criteria for this study. See (Supplementary Figure 1) for the PRISMA flow diagram outlining the study selection process.

### Data Extraction and Quality Assessment

We created two templates for study extractions: one for study characteristics and findings data, and one for risk of bias assessment. The first template included the following: title, authors, year, country, study aim, study design, start date, end date, population description, inclusion criteria, age range, exclusion criteria, type of presentations, type of ED, method of screening, total number of participants, total approached, sample description, screening tool(s) used, definition of a positive, percent positive, factors associated with a positive screening result, and outcomes associated with a positive screening result. Where studies included samples from multiple age groups or settings, we extracted only the data that pertained to the ED and youth. The risk of bias template was based on the NIH Quality Assessment Tool for Observational Cohort and Cross-sectional Studies [https://www.nhlbi.nih.gov/health-topics/study-quality-assessment-tools]. Two reviewers conducted extraction for each article independently using Covidence software, with discrepancies resolved by consensus or a third reviewer.

### Study Synthesis

Due to the heterogeneity of the screening tools and populations identified, the data were unsuitable for a meta-analysis. In this narrative synthesis, we identify the most widely used screening tools and present results for each of these tools: a description of the tool, its definition of a positive screen, where and how they were applied in located studies, prevalence of positive screens, and factors and outcomes associated with a positive screen.

## Results

### Characteristics of Included Studies

A total of 43 papers were deemed eligible for this systematic review, given the aforementioned criteria. The studies are summarized in Table 1. Most studies were conducted in the United States (*n* = 38), three studies in Canada, and one each in the United Kingdom and Australia. Six studies were published before 2010, 20 were published between 2010 and 2019 and 17 papers were published since 2020. Most studies were cross-sectional (*n* = 31) rather than cohort studies (*n* = 12). Almost all of the studies took place in pediatric general EDs (*n* = 33), with a handful of studies taking place in general EDs (*n* = 5), psychiatric EDs (*n* = 3), one pediatric psychiatric ED, and one urgent care center. Sample sizes of adolescent ED patients ranged from 30 (33) to 31,610 (19). Twelve years old was the most common lower age limit for screening (*n* = 12). However, seven studies started screening patients at 8 years of age and other studies conducted screening with patients as young as four (17), five (53), and six (44) years old. Sixteen studies focused only on patients presenting with psychiatric or behavioral chief concerns, while the rest focused on patients presenting with psychiatric or medical/surgical concerns or in a few cases (23, 29, 34), medical/surgical patients only. Patients in studies of psychiatric samples tended to be younger, with most (*n* = 10/16) having a mean age between 13 and 15, as opposed to 14 and 16 (*n* = 18/27) in the general/non-psychiatric studies. Almost every study had more girls than boys (*n* = 37) in their sample, ranging from 39% female (43) to 73% female (18). Most studies had majority White patients but, notably, ten studies had samples with predominantly Black/African American participants. Generally, the quality of included studies was high. Almost all the studies had a clear aim, clearly specified and defined study population, consistency in recruitment, and valid measures. In studies that reported the enrollment rate, most studies (*n* = 27) enrolled more than 50% of those eligible.

**Table 1:** Characteristics of included studies.

**References**	**Country**	**Study design**	**Setting**	**Age range (years)**	**Screening tool**	**Total n**	**Sample description**	**Type of presentation**
Ballard et al., (13)	US	Cohort	PED	8 to 18	RSQ	442	47% female 91% Black/A-A Median age 14 years (IQR 11–15)	Psychiatric only
Ballard et al., (14)	US	Cohort	PED	8 to 18	ASQ	768	53% female 66% Black/A-A Mean age 13.4 years	Psychiatric only
Brent et al., (15)	US	Cohort	PED	12 to 17	ASQ, Death Implicit Association Test, C-SSRS, PHQ-9	1,679	64% female 56% White, 23% Black/A-A, 22% Latinx Mean age 15.1 years (SD 1.6)	Some psych, some med/surg
Burke et al., (16)	US	Cross-sectional	PED	14 to 24	BHS	12,001	65% female 52% Black/A-A, 32% White, 9% Hispanic Mean age 15.79 years (SD 1.40)	All
Cappelli et al., (17)	CAN	Cross-sectional	PED	4 sto 17	HEADS-ED	313	58% female Mean age 14.3 years	Psych only
Cappelli et al., (18)	CAN	Cross-sectional	PED	12 to 17	HEADS-ED	639	73% female Mean age 15.2 years (SD 1.4)	Psych only
Crandal et al., (19)	US	Cross-sectional	PED	12 to 17	C-SSRS	31,610	49% female 45% Hispanic/Latino, 31% White, 6% Asian/Pacific Islander, 4% Black/A-A Mean age: 14.5 (SD 1.9)	Depressed patients only
Cwik et al., (20)	US	Cohort	PED	8 to 21	ASQ	2,466	54% female 67% Black/A-A, 24% White, 10% other/biracial, 3% Hispanic Mean age 13.4 years (SD 2.6)	Psych only
Czyz et al., (21)	US	Cohort	Psych ED	13 to 24	Self-Assessed Expectations of Suicide Risk Scale. C-SSRS	340	58% females 66% White, 20% Black/A-A, 3% Asian, 4% Hispanic, 8% other Mean age 17.6 years (SD 3.3)	Psych only
DeVylder et al., (6)	US	Cohort	PED	8 to 18 years	ASQ	15,003 (4,666 psych, 10,337 all comers)	**Overall**: 53% female 68% Black/A-A; Mean age 14.5 years (SD 3.1) **Selective Sample**: 55% female 68% Black/A-A Mean age 14.0 years (SD 3.1) **Universal Sample**: 52% female 68% Black/A-A Mean age: 14.7 years (SD 3.2)	Part psychiatric (selective phase), part all comers (universal phase)
DeVylder et al., (22)	US	Cohort	PED	8 to 17	ASQ	87	60% female 69% Black/A-A, 22% White Mean age 15.1 years (SD 2.0)	Psychosis patients only
Fein et al., (23)	US	Cross-sectional	PED	14 to 18	BHS-ED	857	56% female Mean age 16.2 years (SD 1.3)	Non-psychiatric
Folse et al., (24)	US	Cross-sectional	ED	12 to 24	RSQ	39	72% female 69% White, 28% Black/A-A Mean age 18 years (SD 3.3)	All
Folse and Hahn, (25)	US	Cross-sectional	ED	12 to 24	RSQ	59	61% female 68% White, 27% Black/A-A, 3% Hispanic Mean age 19 years (SD 2.5)	All
Gipson et al., (26)	US	Cohort	Psych ED	13 to 17	C-SSRS	178	56% female 74% White, 20% Black/A-A, 3% Hispanic, 2% Asian Mean age 15.3 years (SD 1.3)	Psych only
Haroz et al., (27)	US	Cohort	PED	10-18 med 8-18 psych	ASQ	13,420	53% female 63% Black Non-Hispanic, 25% White non-Hispanic, 7% Hispanic, 5% other Mean age 14.3 years	All
Hatkevich et al., (28)	US	Cross-sectional	PED	12 to 17	C-SSRS	5909	59% female 25% Hispanic, 42% non-Hispanic White, 18% non-Hispanic Black/A-A Mean age ~ 15 years	All
Hengehold et al., (29)	US	Cross-sectional	PED	12 to 17	ASQ	3,388	56% female 61% White, 33% Black/A-A Mean age ~14.5 years	Non-psychiatric
Herres et al., (30)	US	Cross-sectional	PED	14 to 24	BHS-ED	3,523	67% female 9% Hispanic, 31% White, 52% Black/A-A Mean age 15.9 years (SD 1.5)	All
Hill et al., (31)	US	Cross-sectional	PED	11 to 21	CSSRS	12,827	59% female 48% Hispanic/Latinx, 27% non-Hispanic White, 19% non-Hispanic Black/A-A, 3% Asiam Mean age 14.5 years (SD 2.2)	All
Hill et al., (32)	US	Cross-sectional	PED	11 to 19	CSSRS	12,401	57% female 48% Hispanic/Latinx, 72% White, 19% Black/A-A, 3% Asian Mean age 14.6 (SD 2.1)	All
Hooper Weatherly, (33)	US	Cohort	Other	14 to 18	CSSRS	30	60% female 73% White, 23% Black/A-A Mean age 15.6 years	Psych only
Hopper et al., (34)	AUS	Cross-sectional	PED	13 to 18	RSQ; SIQ	100	40% female Mean age 14.5 years	Non-psychiatric
Horowitz et al., (35)	US	Cross-sectional	PED	Not reported	RSQ, SIQ, SIQ-JR	144	54% female 49% White, 26% Black/A-A,15% Hispanic Mean age 13.6 years (SD 2.5)	Psych only
Horowitz et al., (10)	US	Cross-sectional	PED	10 to 21	RSQ-Revised, SIQ	156	56% female, 67% Black/A-A, 15% White, 5% Hispanic, 14% mixed/other Mean age 14.6 years (SD 2.8)	Some psych, some general
Horowitz et al., (36)	US	Cross-sectional	PED	10 to 21	ASQ, SIQ, SIQ JR	524	57% female 50% White, 30% Black/A-A, 9% Hispanic, 2% Asian, 9% other Mean age 15.2 years (SD 2.6)	All
Horwitz et al., (37)	US	Cohort	Psych ED	15 to 24	C-SSRS	473	53% female 69% White, 17% Black, 5% Asian, 2% Hispanic, 7% Multiracial Mean age 19.4 years (SD 2.9)	Psych only
Kennedy et al., (38)	US	Cross-sectional	Ped psych/BH ED	8 to 17	Childhood Acuity of Psychiatric Illness (CAPI) has a suicide item	553 (for CAPI)	57% female Mean age 14.0 years (SD 2.36)	Psych only
King et al., (39)	US	Cross-sectional	ED	13 to 17	SIQ JR	298	50% Females 83% White, 16% Black/A-A, 2% American Indian /Alaskan Native, 3% Asian, 5% Hispanic. Mean age 15.0 years (SD 1.4)	All
King et al., (40)	US	Cross-sectional	PED	13 to 17	SIQ JR	245	53% female 80% White, 22% Black/A-A, 10% American Indian or Alaskan Native, 3% Asian, 6% Hispanic Mean age 15.3 years (SD 1.4)	All
King et al., (41)	US	Cohort	PED	12 to 17	C-SSRS, ASQ, Computerized Adaptive Screen for Suicidal Youth (CASSY)	Study 1: 6,536 Study 2: 4,050	Study 1: 59% female 48% white, 22% Black/A-A, 25% Hispanic Mean age 15.0 (SD 1.7). Study 2: 64% female, 56% White, 19% Black/A-A, 25% Hispanic Mean age 15.0 years (SD1.7)	All Presentations for Study 1 Enriched with psychiatric presentations for Study 2
Lantos et al., (42)	US	Cross-sectional	PED	12 to 24	ASQ	T1: 19,265 T2: 9,984	54% Female 60% White, 18% Black/A-A Median age 15 years (IQR 13–16)	All
Lanzillo et al., (43)	US	Cross-sectional	PED	10 to 12	ASQ, SIQ-Jr	79	39% female 49% White Mean age 11.2 years (SD 0.8)	Some psych, some general
Latif et al., (44)	US	Cross-sectional	PED	6 to 17	C-SSRS	879	55% female 63% Non-Hispanic Black/A-A Mean age 13.4 years (SD 2.8)	Psych only
Manning et al., (45)	UK	Cross-sectional	PED	10 to 19	CYP-MH SAPhE, C-SSRS	163	66% female 87% White British/Irish Mean age 14.3 years (SD 1.8)	Some psych, some general
Patel et al., (46)	US	Cross-sectional	Urgent care	12 and older	2-item screener (life NOT worth living? have you wanted to kill yourself?); CSSRS	4,786	56% female 69% White, 13% Black/A-A, 9% Hispanic Age: 56% between 12–14 years old 43% between 15–19 years old	All
Powell et al., (47)	US	Cross-sectional	PED	10 to 21	SIQ, SIQ-JR ASQ	522	57% female 50% White, 30% Black/A-A, 9% Hispanic, 7% Other Mean age 15.3 years (SD 2.6)	All
Roaten et al., (48)	US	Cross-sectional	ED	12 to 17, then 10 to 17	ASQ	9,577	6% female 72% White Hispanic, 5% White non-Hispanic, 21 Black non-Hispanic Mean age 14.9 years (1.8 SD)	All
Rutman et al., (49)	US	Cross-sectional	PED	12 to 17	SIQ SIQ-JR	78	60% female 53% White, 35% Hispanic, 5% Black/A-A, 8% other	Depressed patients
Stanley et al., (50)	US	Cross-sectional	PED	10 to 21	SIQ/SIQ-JR, RSQ single item (Q4)	524	57% female 50% White, 30% Black/A-A, 9% Hispanic Mean age 15.2 years (SD 2.6)	All
Stanley et al., (51)	US	Cross-sectional	PED	10 to 21	ASQ, SIQ, SIQ-JR	524	57% female 50% White, 30% Black/A-A, 9% Hispanic Mean age 15.2 years (SD 2.6)	All
Sullivant et al., (52)	US	Cross-sectional	PED	12 to 21	ASQ (C-SSRS but not in ED)	138,598 overall (# in ED not specified)	Not specified	All
Williams et al., (53)	US	Cross-sectional	ED	5 to 18	Crisis Assessment Tool (CAT)	225	53% female Mean age 14.1 years (SD 2.7)	Psych only

*PED, Pediatric Emergency Department; ED, Emergency Department*.

*ASQ, Ask Suicide Questions; SIQ, Suicidal Ideation Questionnaire; CSSRS, Columbia Suicide Severity Rating Scale; BHS, Behavioral Health Screen; RSQ, Risk of Suicide Questionnaire; PHQ, Patient Health Questionnaire; HEADS-ED, Home, education, activities/peers, drugs/alcohol, suicidality, emotions/behavior, discharge resource*.

### Screening Tools

The most common screening tools being tested or implemented were the Ask Suicide-Screening Questions (ASQ) (*n* = 15), the Columbia-Suicide Severity Rating Scale (C-SSRS) (*n* = 12), the Suicidal Ideation Questionnaire (SIQ) (*n* = 10), and the Risk of Suicide Questionnaire (RSQ) (*n* = 7) (Table 2; several studies used more than one screener). Several studies used less well-established suicide screening tools or used general mental health screeners that included a suicide item (see “Other Suicide Screening Tools” below). Of note, some studies used more than one screening tool. Most studies involved the routine administration of the screener by clinical staff (*n* = 27), most often by nurses (*n* = 13). The other studies relied on research staff to administer the screener (*n* = 16). Many of the studies asked that a parent or caregiver not be present while the screener was administered. Where modality was specified (*n* = 30), most screeners were administered verbally (*n* = 20) or on a computer/tablet (*n* = 8), while two studies used paper-based screening.

**Table 2:** Suicide screening tools used most often with pediatric emergency patients.

**Screening Tool**	**Studies**	**Content**	**Number of Items**	**Percent positive**
ASQ	(6, 14, 15, 20, 22, 27, 29, 36, 41–43, 47, 48, 51, 52)	1) In the past few weeks, have you wished you were dead? 2) In the past few weeks, have you felt that you or your family would be better off if you were dead? 3) In the past week, have you been having thoughts about killing yourself? 4) Have you ever tried to kill yourself? If the patient responds “yes” to Question 4, one must inquire how and when the attempt occurred. If the patient answers yes to any of the first four questions, a fifth question, (Q5) “Are you having thoughts of killing yourself right now?”	4 (5 if positive)	All patients: 8% (6) to 29% (47). Psych patients: 36% (20) to 66% (47). Medical/surgical patients: 3% (48) to 10% (36)
CSSRS	(15, 19, 21, 26, 28, 31–33, 37, 41, 44, 52).	Level of recent ideation in terms of intensity, frequency, duration, controllability, deterrents, reasons, and behavior pattern	Up to 7 items	All patients: 19% (Hill et al., (32)) Psych patients: 66% (33) to 86% (45)
SIQ/SIQ JR	(10, 34–36, 39, 40, 43, 47, 49–51)	Self-reported screening tool that assesses suicidal ideation on a 7-point scale with statements about frequency of suicidal thoughts or risk factors. Some critical items directly assess serious self-destructive behavior.	SIQ: 30 (9 critical items). SIQ JR: 15 (6 critical items)	All patients: 4% (40) to 29% (47). Psych patients: 40% (10) to 66% (47). Medical/surgical patients: 0% (34) to 10% (47)
RSQ	(10, 13, 24, 25, 34, 35, 50)	(Q1) Are you here because you tried to hurt yourself? (Q2) In the past week, have you been having thoughts about killing yourself? (Q3) Have you ever tried to hurt yourself in the past (other than this time)? (Q4) Has something very stressful happened to you in the past few weeks (a situation that was very hard to handle)?	4	All patients: 28% (24) to 51% (25). Psych patients: 48% (13). Medical/surgical patients: 22% (34)

#### Ask Suicide-Screening Questions (ASQ)

The ASQ is a screening tool that was developed by Horowitz et al. (36) for patients aged 10 to 21 years old. Fifteen studies tested or implemented the ASQ. In development, the team used the SIQ as the criterion standard and studied 17 candidate screening questions for evaluating suicide risk in young patients based on risk factors for suicide in adolescents, including suicide attempt history, suicidal ideation, depression, hopelessness, substance abuse, and social isolation. Against the SIQ, the ASQ had a sensitivity of 96.9 and specificity of 87.6. The final tool is comprised of four simple questions to evaluate suicide risk in youth populations: (1) In the past few weeks, have you wished you were dead? (2) In the past few weeks, have you felt that you or your family would be better off if you were dead? (3) In the past week, have you been having thoughts about killing yourself? (4) Have you ever tried to kill yourself? If the patient responds “yes” to Question 4, one must inquire how and when the attempt occurred. If the patient answers yes to any of the first four questions, a fifth question, (Q5) “Are you having thoughts of killing yourself right now?”, is asked to aim to assess the acuity of current risk. A “yes” response to any of the four questions (Q1-Q4) indicates a positive screen. In more recent analyses, the importance of a patient choosing the “no response” option has become evident: patients who intentionally endorsed “no response” (as opposed to “yes” or “no”) were of a similar profile to those who endorsed “yes” (29), such that a positive screen is often operationalized as a “yes” or “no response” to any item and a negative screen is “no” to all items. In our review, we found that the tool was most often administered verbally in pediatric EDs, typically in samples of patients with both medical/surgical and psychiatric complaints (*n* = 11).

In studies of patients with all chief complaints (including psychiatric and medical/surgical), the positivity rate ranged from 8% (6) to 29% (47). In studies that only included patients with psychiatric chief complaints (or studies that included a breakdown of solely psychiatric presentations), positivity rates ranged from 36% (20) to 66% (47). In contrast, in studies with patients with only medical/surgical complaints or studies that included a breakdown of solely non-psychiatric presentations, the positivity rates ranged from 3% (48) to 10% (36). A few papers explored the ASQ's effectiveness in screening younger populations, as young as 8 years old (6, 14, 20, 22, 27). Those who screen positive on the ASQ tend to be more often female (6, 14, 20, 22, 29, 42, 47), older (14, 20, 22, 29, 42); and, in “all comer” samples, to present with a psychiatric/suicide-related complaint (6, 22, 43). Being Black/African- American was protective in some studies (14, 42) and a risk factor in others (20, 22, 29). Those with a positive ASQ result were more likely to be admitted (6, 14, 22, 48) and more likely to re-present with to the ED (6), especially with a suicide-related complaint (14, 20, 27). In studies that examined criterion validity, the ASQ had acceptable sensitivity (60-93%) and specificity (43–92%) in predicting future attempts (6, 14, 20, 27, 41). It also performed well against the longer SIQ with a sensitivity of 97% and specificity of 88% (36).

#### Columbia-Suicide Severity Rating Scale (CSSRS)

The C-SSRS is a measure used to identify and assess individuals at risk for suicide, with a special focus on ascertaining levels of recent and lifetime ideation and behavior. Evidence around its psychometric properties has been mixed (54–58). Twelve studies located in the current review tested or implemented a version of the Columbia Suicide Severity Rating Scale (C-SSRS).

The screener version [used in Hooper Weatherly (33), Crandal et al. (19), Latif et al. (44), Brent et al. (15), Hill et al. (32), Hill et al. (31)] is up to seven items in length. Patients are asked in the past month “have you wished you were dead or wished you could go to sleep and not wake up” (Yes/No) or “actually had any thoughts of killing yourself?” If the patient endorses the second question, they are asked if they have: been thinking about how they might do this; had these thoughts and had some intention of acting on them; or started to work out or worked out the details of how to kill themselves and intend to carry out the plan. Finally, all patients are asked if they have ever “done anything, started to do anything, or prepared to do anything” to end their life, and if so whether that occurred in the past 3 months. A positive is usually defined as a yes to any recent ideation or yes to suicide attempt, though one paper excluded passive ideation from its definition of a positive (33). The full C-SSRS assessment (used in King et al. (41), Horwitz et al. (37), Manning et al. (45), Gipson et al., (26), Czyz et al., (21)) is more detailed: in addition to assessing the level of recent ideation, it assesses ideation at the patient's worst point, as well as assessing its intensity, frequency, duration, controllability, deterrents, and reasons. For behavior, the full assessment breaks behavior into actual, interrupted attempt, aborted, and preparatory behavior, and its lethality.

The C-SSRS was administered to a variety of patients: six studies applied the tool in a psychiatric sample and six applied it to a mixed medical/psychiatric sample. The youngest patients who received the CSSRS were aged six (44), but most studies' lower age limit was at least 12 years of age. Most studies implemented the C-SSRS into routine clinical workflow by nurses, behavioral health providers, and medical assistants. In the psychiatric samples, positivity ranged from 66% (33) to 86% (45). Some studies reported more granular detail on psychiatric positives: for example (44), reported that 40% of patients in their psychiatric sample were deemed “high risk” on the C-SSRS screener and Gipson et al. (26) reported that 45% of psychiatric patients had recent active ideation and 30% had a lifetime attempt. In “all patient” samples, there was still a significant positivity rate on the C-SSRS, reaching as high as 19% (31). Hatkevich et al. (28) used the attempt item from the C-SSRS to examine how wording affected patients' likelihood of screening positive. Comparing a directly phrased question asking about “suicide attempt” to an indirectly phrased question providing the definition of an attempt, they found that 10% of patients endorsed both, 3% endorsed the directly phrased questions, and 3% endorsed the indirectly phrased one. Patients who were positive on the C-SSRS were more often female (19, 26, 28, 31, 32, 37, 45), older (19, 32, 44), and less likely to be Hispanic (19, 31, 32). In studies with psychiatric samples, rates of admission (33, 44) were higher among those who screened positive. Ideation intensity (26, 37) and severity (21) on the C-SSRS was associated with future suicide-related visits, showing that the tool has predictive validity in this pediatric emergency patient population.

#### Suicidal Ideation Questionnaire (SIQ)

Eleven studies tested or implemented the SIQ. The SIQ is a 30-item self-reported screening tool that was developed by Reynolds (59) for high school students in grades 10-12 (and the 15-item SIQ-JR for students in grades 7-9) to screen for suicide risk (60). The tool has acceptable psychometric properties (61, 62) and was quickly applied to healthcare settings. The SIQ used to screen patients 15 years and older and the SIQ-JR for those patients 10 to 15 years old. The tool was most often administered via a written self-reported questionnaire in pediatric EDs (*n* = 10), typically in samples of patients with both medical/surgical and psychiatric complaints (*n* = 8). Most studies that included the SIQ (*n* = 7) were applying it as a gold standard against which to test other, shorter, screening tools.

The screening tool assesses suicidal ideation on a 7-point scale with statements about frequency of suicidal thoughts or risk factors; for example, a patient would rank “I thought it would be better if I was not alive” on a scale from “I never had this thought” (0) to “almost every day” (7). These point scales are added up to give rise to a score between 0 and 180 for the SIQ, or 0–90 for the SIQ-JR. A score of 41 or greater on the SIQ, a score of 31 or greater on the SIQ-JR, or an endorsement of a recent suicide attempt constitute a positive screen and warrant further psychiatric evaluation. Nine critical items (six on the SIQ-JR) directly assess serious self-destructive behavior, with endorsement of three or more of these items (two on the SIQ-JR) constituting a positive screen for suicidal ideation, regardless of total score (10).

In samples that included patients with any chief complaint, the positivity rate of the SIQ ranged from 4% (40) to 29% (47). In studies that only sampled patients with psychiatric chief complaints, or studies that included a breakdown of solely psychiatric presentations, positivity rates ranged from 40% (10) to 66% (47). In contrast, in studies with patients with only medical/surgical complaints, or studies that included a breakdown of solely non-psychiatric presentations, the positivity rates ranged from 0% (34) to 10% (47). Those who screened positive on the SIQ tended to more often be female (39, 47), and more often presenting with psychiatric complaints: in universal screening studies, the proportion of positive screens that were positive ranged from 77% (10, 47) to 87% (43). King et al. (39) showed that the SIQ had good concurrent validity with a measure of hopelessness, a risk fact for suicide.

#### Risk of Suicide Questionnaire (RSQ)

Seven studies tested or implemented the RSQ. The RSQ is an older four-item screening tool that was developed by Horowitz et al. (35) to be administered by triage nurses in EDs to children between the ages of 8–21 years old. The tool was originally developed from 14 potential screening questions from several sources, which were validated among several pediatric clinicians and mental health specialists, as well as a sample of pediatric psychiatric patients and nonpatients. The final tool includes four questions and was validated cross-sectionally using a “gold standard” assessment comparison with the SIQ. The tool was most often administered via a verbal questionnaire administered by research staff in pediatric EDs (*n* = 5), typically in samples of patients with both medical/surgical and psychiatric complaints (*n* = 4).

The RSQ asks four questions: (Q1) Are you here because you tried to hurt yourself? (Q2) In the past week, have you been having thoughts about killing yourself? (Q3) Have you ever tried to hurt yourself in the past (other than this time)? (Q4) Has something very stressful happened to you in the past few weeks (a situation that was very hard to handle)? A positive screen is defined as answering “yes” to any question. Folse et al. (24) used a broader definition of positive to include “no response” accompanied by nonverbal behaviors of concern.

The positivity rate based across studies ranged from 28% (24) to 51% (25) of “all comers”. Only one study sampled solely patients with psychiatric chief complaints and reported an overall positivity value, of 48% (13). In contrast, one study sampled solely patients with non-psychiatric complaints and found a positivity rate of 22% (34). Positive screens were much more likely to have a psychiatric presenting complaint (10, 50). One study (50) focused only on the fourth question of the RSQ (“Has something very stressful happened to you in the past few weeks?”) and found that nearly 80% of patients screened endorsed this item, a very high rate of positivity, leading to concerns about the specificity of this tool. It is notable that Hopper et al. (34) found that one-fifth of their non-psychiatric sample screened positive on the RSQ while screening negative on the SIQ, suggesting an issue with false positives. Where criterion validity was reported, the RSQ was found to have high sensitivity (50–98%) but low specificity (37–79%) against the longer SIQ (25, 34, 35). Folse and Hahn (25) concluded that the RSQ had inadequate reliability in their sample.

#### Other Suicide Screening Tools

We located several less-frequently used suicide screening tools, as well as some general mental health screeners that included suicide-related items.

Brent et al. (15) applied the Death Implicit Association Test with a cohort of medical and psychiatric ED patients aged 12–17 years. The IAT was predictive of 3-month attempts in a risk-saturated sample, with an AUC of 0.59 but performed better in patients who were non-suicidal at baseline (AUC = 0.67). Czyz et al. (21) applied the Self-Assessed Expectations of Suicide Risk Scale, which consists of three questions on a 0 (not at all confident) to 10 (extremely confident) scale. It rates the patient's confidence that they will: not attempt suicide; be able to keep from killing themselves if serious suicidal thoughts occur; and tell someone about suicidal thoughts if they occur. In a cohort of 340 13- to 24-year-olds visiting a psychiatric ED, the area under the curve (AUC) for a future suicide attempt was 0.79 for the full Scale and 0.80 for the second item on its own. The optimal cut-off for that item was 6.5/10, which generated a sensitivity of 79% and specificity of 76%; this item was also the strongest predictor of time-to-suicide attempt. King et al. (41) developed and validated the Computerized Adaptive Screen for Suicidal Youth in two larges samples of pediatric ED patients. They found high predictive accuracy of the tool for future suicide attempts, with areas under the curve of 0.87–0.89. It was also brief: the mean number of items administered was 11. Manning et al. (45) tested the Children and Young People-Mental Health Self-harm Assessment in Pediatric healthcare Environments (CYP-MH SAPhE) in a sample of 10–19 year-old psychiatric and medical ED and inpatients. The tool focuses on in-situ risk, with questions like “Right now, do you wish you were dead?” (endorsed by 42% of psychiatric patients, 0% of medical patients) and “At the moment, do you have a plan to end your life?” (endorsed by 21% of psychiatric patients, 0% of medical patients). The tool demonstrated high reliability, congruence with the CSSRS, and predictive validity. In a pediatric urgent care center, Patel et al. (46) applied a two-item screener to all patients aged 12 and older: “In the past week including today, have you felt like life is NOT worth living?” and “In the past week including today, have you wanted to kill yourself?”. Two per cent of their patients screened positive and the tool was congruent with the CSSRS. Patients screening positive were more likely to be female and less likely to be White.

There were several studies that presented general mental health screeners that included suicide-related items. Three studies used the Behavioral Health Screener (BHS): one used the full tool (16) and two used the abbreviated ED version (BHS-ED; (23, 30)). Several BHS items assess the presence of suicidal thoughts and behaviors (“Have you felt that life is not worth living?”; “Have you thought about killing yourself?”; “Have you made a plan to kill yourself?”; and “Have you tried to kill yourself?”) over the patient's lifetime and past week. In a sample of 14- to 24-year-old “all comers,” Burke et al. (16) found lifetime attempt in 9% of ED patients and past-week attempt in 1.7%. Using the same age range, Herres et al. (30) found lifetime active suicidal ideation in 20% of patients, lifetime attempt in 9% of patients, and past-week attempt in 1.5% of patients. In a group of non-psychiatric ED patients aged 14–18, Fein et al. (23) found that 6% had made a lifetime attempt and 0.7% had a past-week attempt. The HEADS-ED tool was used in two studies (17, 18), both of which enrolled psychiatric patients only. The tool has one item on suicidality with three levels: none, ideation only, gesture/plan. Cappelli et al. (17) found that 25% of patients were positive for gesture/plan and 78% were positive for ideation/gesture/plan. Those who endorsed suicidality were more likely to receive a consult and admission. In another psychiatric sample, Cappelli et al. (18) reported 31% positivity for gesture/plan and 70% positive for ideation/gesture/plan: again, these patients were more likely to receive consult and admission. The Childhood Acuity of Psychiatric Illness scale also has an item assessing levels of suicidal ideation/gesture from low (none/mild) to high (moderate/severe): in a psychiatric sample, Kennedy et al. (38) found that 8% of 8- to 11-year-olds were in the high category compared to 32% of 12- to 17-year-olds. Finally, the Crisis Assessment Tool is 38-item measure that assesses 6 domains, including child risk behaviors like acute suicide risk, from 0 (no evidence that the item requires action) to 3 (severe or immediate need for action). Williams et al. (53) found that 57% of their psychiatric sample had acute suicide risk at level 3 (severe concern). These patients were more likely to be female and to be admitted.

## Discussion

Suicide rates are increasing among youth (2), and EDs provide a potential venue for detection and management of suicide risk. We located four commonly used screening tools for suicidality in pediatric ED patients (the ASQ, CSSRS, SIQ, and RSQ), as well as several less widely researched tools. The studies were generally of good quality, and the amount of research on this topic appears to have increased significantly in the past decade. The vast majority of research was conducted in the United States, which may reflect the increasing focus on screening from accreditation and advocacy organizations (44, 63) in that country.

Most of the screeners were identified were brief and feasible to implement in routine care. They uncovered suicide risk in about half of psychiatric samples and up to 20% of medical/surgical patients. This differential finding reflects the strong association between psychiatric morbidity and suicide (64), while also showing that some presentations for non-psychiatric reasons may harbor a surprising level of suicide risk (4). The RSQ seemed to be associated with very high positivity rates, leading to concerns about false positives (i.e. patients who screen positive on the tool who are not actually experiencing suicidality) and unmanageable numbers of consults. Many of the false positives on the RSQ were likely due to an item about recent stressors and this presumably is the reason for the decline of that tool in the last decade. It was notable that across several screening tools, being female was associated with a positive screening result. Although males are more likely than females to die by suicide, females are more likely to both ideate about and attempt suicide (65). At a time when youth suicide rates among Black youth are increasing (66), it was promising to see that a significant number of studies included majority Black/African-American patients.

The COVID-19 pandemic has been associated with a significant increase in rates of suicidality in youth (67, 68). Although a significant number of the studies in our review were published after 2020, just three of the studies included data that was collected during the pandemic. Two of these studies showed an increase in the positive suicide screening rate during the pandemic compared to before (31, 42); the other study (19) did not compare rates before and after but showed a dramatic dip in the number of screenings administered during January and April 2020. As well as showing the disruption the pandemic caused to usual care, these findings support the earlier finding that rates of suicidality among youth appear to have increased during the pandemic.

Although screening for suicidality with a single screening tool may be efficient, the risk factors (and protective factors) for suicide are complex and dynamic: it should be noted that detecting suicide risk with a simple screener is merely the first step in understanding and managing suicide risk clinically in youth. Once risk is detected using a screener, a more detailed suicide risk assessment by a trained behavioral health provider should address a range of contributing factors, such as: behavioral health morbidity; past, recent and current suicidality; stressors and adversity; impulsivity and aggression; social supports and coping mechanisms; treatment engagement; access to lethal means; and feelings of hopelessness, shame, and guilt. Once the clinician has a better sense of the patient's needs and resources, they may tailor an intervention to that patient's circumstances. Clinical interventions shown to prevent subsequent future suicidal behavior in youth include Dialectical Behavior Therapy for Adolescents and Cognitive Behavioral Therapy (69).

The current systematic review has several limitations to bear in mind. Given our suicide-focused search strategy, we may have failed to identify additional general mental health screeners that include suicide-related items. We focused on the ED setting, so these findings may not be applicable to other care settings. We also limited our review to studies published in English, which may affect the generalizability of the findings to international settings. Finally, because of the heterogeneity of tools and analyses, we were not able to conduct a meta-analysis of the results. However, we believe that this systematic review will be useful to those seeking to implement suicide screening with the pediatric emergency patients.

When selecting a suicide screening tool for pediatric emergency settings, it is important to consider several factors that may impact its success. First, it is important to decide whether to implement the tool universally (with all patients regardless of presenting complaint) or only with patients with psychiatric presenting complaints. The former approach may be more resource-intensive than the latter but will allow for the detection of suicide risk that might otherwise be missed (6). In choosing a screening tool, it is important to select one that is supported by research but is also feasible within a busy ED setting: it should be brief and easy for non-specialists to administer (5). Computer-based approaches allow for standardization and privacy but require hardware and sometimes proprietary software; verbal administration has the potential for a more impactful interpersonal connection and disclosure but can be stymied by negative framing (70) and poor fidelity (71). It is also important to develop a protocol around how to support patients who screen positive in terms of assessment, intervention, and follow-up. If resources are very limited, an ED might choose a tool with a high threshold to minimize the number of positives, while other EDs may prefer a screening tool that detects very low levels of suicidality to avoid missing a patient at risk. In this review, we found that there are several well-supported tools available for screening suicide risk in young ED patients, and the tool chosen may be informed by the needs and resources of the department.

## Data Availability Statement

The original contributions presented in the study are included in the article/Supplementary Material, further inquiries can be directed to the corresponding author.

## Author Contributions

AS, RR, and CL selected the articles and completed the quality assessment, analysis of results, writing of the introduction, results, discussion, contributed and collaborated to design tables, writing of manuscript, and conclusions. BB designed the search strategies and filters with use of the Covidence platform and wrote the majority of the methods section. EB provided conceptual input throughout the entire research process and edited the manuscript. All authors reviewed and revised this writing and approved the submitted version.

## Funding

This research was funded by the Department of Emergency Medicine at the University of Massachusetts Chan Medical School.

## Conflict of Interest

The authors declare that the research was conducted in the absence of any commercial or financial relationships that could be construed as a potential conflict of interest.

## Publisher's Note

All claims expressed in this article are solely those of the authors and do not necessarily represent those of their affiliated organizations, or those of the publisher, the editors and the reviewers. Any product that may be evaluated in this article, or claim that may be made by its manufacturer, is not guaranteed or endorsed by the publisher.
