# Supplementary figures and images for: Suicide Screening Tools for Pediatric Emergency Department Patients: A Systematic Review
Source: Front Psychiatry. 2022 Jul 12;13:916731. doi: 10.3389/fpsyt.2022.916731 (PMC9314735; doi:10.3389/fpsyt.2022.916731)

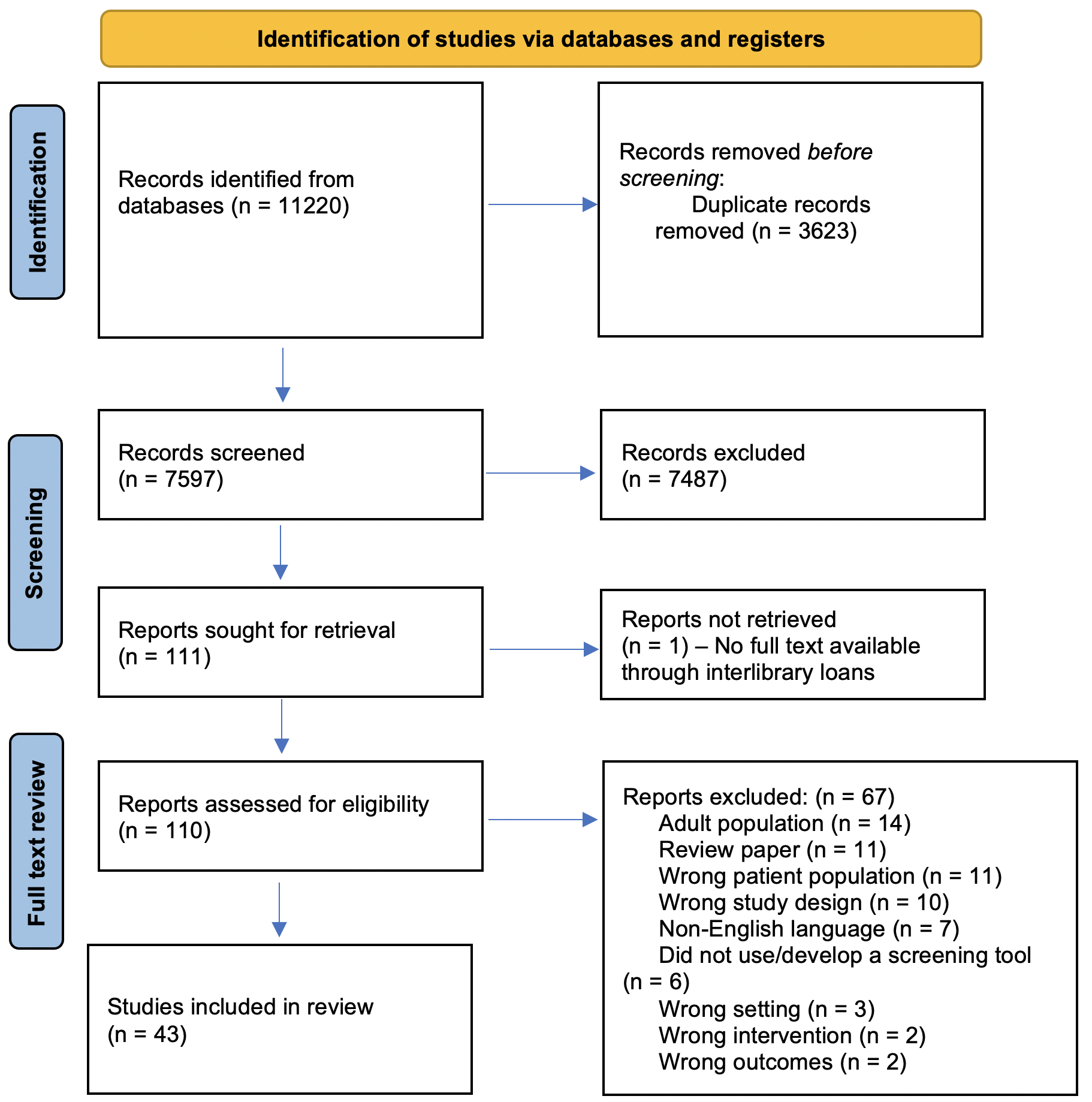

Supplement: Supplementary file 1 [file Image_1.jpg]
