# Supplementary material for: Suicide Screening Tools for Pediatric Emergency Department Patients: A Systematic Review
Source: Front Psychiatry. 2022 Jul 12;13:916731. doi: 10.3389/fpsyt.2022.916731 (PMC9314735; doi:10.3389/fpsyt.2022.916731)
Supplement: Supplementary file 2 [file Table_1.docx]

**Supplementary Table 1:** Full Ovid Medline Search Strategy

| 1. | exp Self-Injurious Behavior/ or (((self or oneself or themsel*) adj3 (burn* or destructive or harm* or injur* or mutilat* or violen* or wound*)) or selfharm* or selfinjur* or NSSH or NSSI or suicid* or parasuicid* or (fatal adj3 attempt*) or (self adj3 (kill* or poison*)) or SIB or automutilat* or cutting).ti. | |
| --- | --- | --- |
| 2. | Emergency Medical Services/ or exp Emergency Medicine/ or exp Emergency Service, Hospital/ or Emergency Services, Psychiatric/ or Emergency Treatment/ or Triage/ |  |
| 3. | ((emergenc* adj3 (care or center* or centre* or department* or medic* or room* or service* or unit* or therap* or treatment* or ward*)) or ED or EDs or ER or ERs or emergicenter* or (trauma* adj3 (center* or centre* or unit*)) or triage*).ti,ab. |  |
| 4. | 2 or 3 |  |
| 5. | Mass Screening/ or exp Psychiatric Status Rating Scales/ or Risk Assessment/ or Self Report/ or "Surveys and Questionnaires"/ |  |
| 6. | (assess* or detect* or evaluat* or identif* or instrument* or intake or measure* or questionnaire* or scale* or screen* or survey* or test* or tool* or (mental adj3 status) or (risk adj3 analys*) or (self adj3 report*)).ti,ab. |  |
| 7. | 5 or 6 |  |
| 8. | 1 and 4 and 7 |  |
